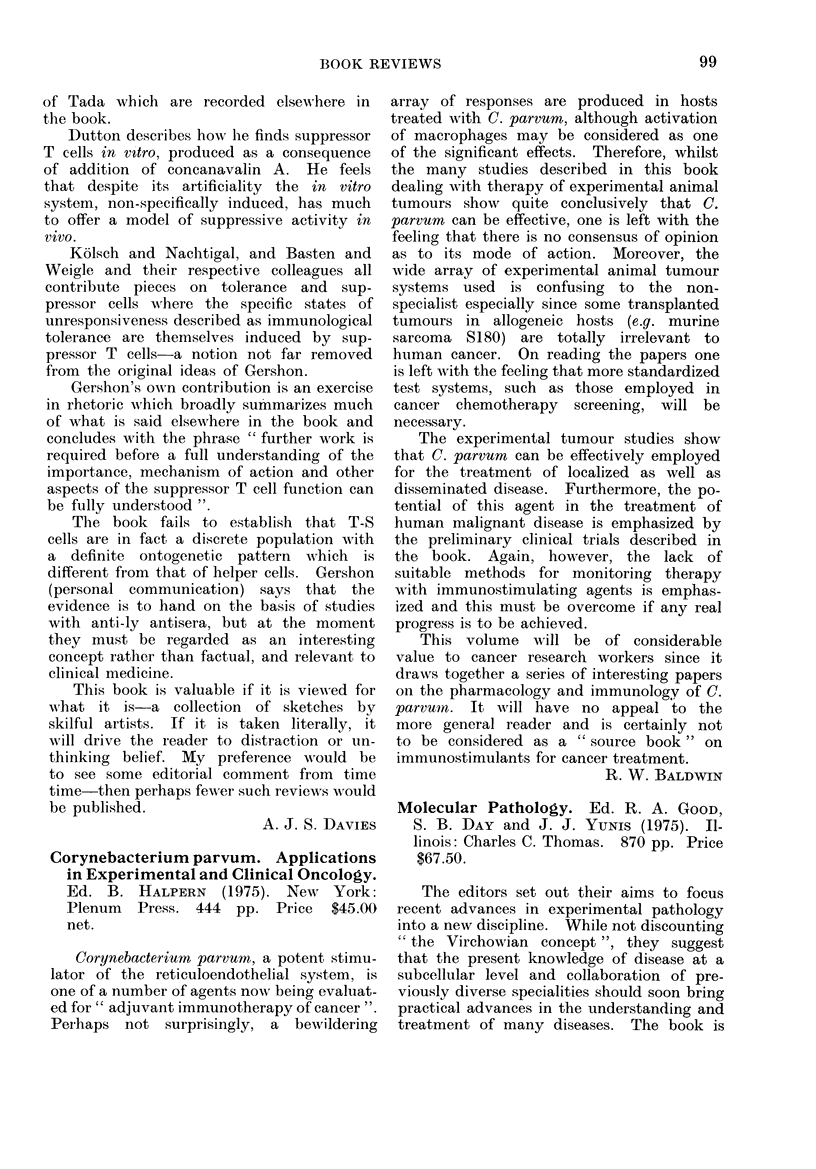# Corynebacterium parvum. Applications in Experimental and Clinical Oncology

**Published:** 1976-07

**Authors:** R. W. Baldwin


**Corynebacterium parvum. Applications in Experimental and Clinical Oncology.** Ed. B. HALPERN (1975). New York: Plenum Press. 444 pp. Price $45.00 net.

*Corynebacterium parvum*, a potent stimulator of the reticuloendothelial system, is one of a number of agents now being evaluated for " adjuvant immunotherapy of cancer ". Perhaps not surprisingly, a bewildering array of responses are produced in hosts treated with *C. parvum*, although activation of macrophages may be considered as one of the significant effects. Therefore, whilst the many studies described in this book dealing with therapy of experimental animal tumours show quite conclusively that *C. parvum* can be effective, one is left with the feeling that there is no consensus of opinion as to its mode of action. Moreover, the wide array of experimental animal tumour systems used is confusing to the non-specialist especially since some transplanted tumours in allogeneic hosts (*e.g.* murine sarcoma S180) are totally irrelevant to human cancer. On reading the papers one is left with the feeling that more standardized test systems, such as those employed in cancer chemotherapy screening, will be necessary.

The experimental tumour studies show that *C. parvum* can be effectively employed for the treatment of localized as well as disseminated disease. Furthermore, the potential of this agent in the treatment of human malignant disease is emphasized by the preliminary clinical trials described in the book. Again, however, the lack of suitable methods for monitoring therapy with immunostimulating agents is emphasized and this must be overcome if any real progress is to be achieved.

This volume will be of considerable value to cancer research workers since it draws together a series of interesting papers on the pharmacology and immunology of *C. parvum*. It will have no appeal to the more general reader and is certainly not to be considered as a " source book " on immunostimulants for cancer treatment.

R. W. BALDWIN